# Analyses of *POL30* (PCNA) reveal positional effects in transient repression or bi-modal active/silent state at the sub-telomeres of *S. cerevisiae*

**DOI:** 10.1186/s13072-023-00513-7

**Published:** 2023-10-19

**Authors:** Safia Mahabub Sauty, Krassimir Yankulov

**Affiliations:** Department of Molecular and Cellular Biology, University of Guelph, Guelph, ON N1G2W1 Canada

**Keywords:** *POL30*(PCNA), CAF1, *ASF1*, *RRM3*, Chromatin, Gene silencing

## Abstract

**Background:**

Classical studies on position effect variegation in *Drosophila* have demonstrated the existence of bi-modal Active/Silent state of the genes juxtaposed to heterochromatin. Later studies with irreversible methods for the detection of gene repression have revealed a similar phenomenon at the telomeres of *Saccharomyces cerevisiae* and other species. In this study, we used dual reporter constructs and a combination of reversible and non-reversible methods to present evidence for the different roles of PCNA and histone chaperones in the stability and the propagation of repressed states at the sub-telomeres of *S. cerevisiae*.

**Results:**

We show position dependent transient repression or bi-modal expression of reporter genes at the *VIIL* sub-telomere. We also show that mutations in the replicative clamp *POL30* (PCNA) or the deletion of the histone chaperone CAF1 or the *RRM3* helicase lead to transient de-repression, while the deletion of the histone chaperone *ASF1* causes a shift from transient de-repression to a bi-modal state of repression. We analyze the physical interaction of CAF1 and *RRM3* with PCNA and discuss the implications of these findings for our understanding of the stability and transmission of the epigenetic state of the genes.

**Conclusions:**

There are distinct modes of gene silencing, bi-modal and transient, at the sub-telomeres of *S. cerevisiae*. We characterise the roles of CAF1, *RRM3* and *ASF1* in these modes of gene repression. We suggest that the interpretations of past and future studies should consider the existence of the dissimilar states of gene silencing.

**Supplementary Information:**

The online version contains supplementary material available at 10.1186/s13072-023-00513-7.

## Introduction

Positional effect variegation is caused by the proximity of a gene to a heterochromatin boundary and is characterised as a stochastic Active/Silent mode of expression. In *Drosophila melanogaster*, this effect is readily observed by the variegated expression of the *white*^*V*^ gene [1]. Seemingly similar stochastic Active/Silent states of the expression of reporter genes has been characterised at the telomeres of *S. cerevisiae* and other model organisms [2].

The Proliferating Cell Nuclear Antigen (PCNA, *POL30*) forms a homo-trimeric sliding clamp which acts at the core of the eukaryotic replisome [3]. PCNA is highly conserved and interacts with numerous proteins, including the leading and lagging strand DNA polymerases, DNA repair polymerases, DNA ligase, and the Fen1p flap-endonuclease. The function of PCNA in DNA replication and repair had been the focus of outstanding reviews [2–4] and will not be discussed here. Both human and yeast PCNA directly interact with Chromatin Assembly Factor-1 (CAF-1), a histone chaperone required for replication-coupled chromatin assembly in vitro [7, 8]. In *S. cerevisiae*, mutations in *POL30* lead to loss of heterochromatin-mediated gene silencing [9–12], thus revealing a direct role in the maintenance and transmission of chromatin structure. Three *POL30* mutations, *pol30-6*, *pol30-8* and *pol30-79* [9, 13], were pivotal in providing details about this role of PCNA. These three alleles have double alanine substitutions at residues Asp 41 and Asp 42 in *pol30-6*, residues Arg 61 and Asp 63 in *pol30-8*, and residues Leu 126 and Ile 128 in *pol30-79*. On the three-dimensional trimeric structure of PCNA, the *pol30-6* mutation is located at the central loop, *pol30-8* is located at the tip of a domain bulge, and *pol30-79* is located at the interdomain connecting loop [9].

At the *VIIL* telomere, the* pol30-6*, *pol30-8* and *pol30-79* alleles lead to a significant loss of gene silencing as detected by the insertion of *URA3* and the measurement of the sensitivity of the cells to 5-Fluoroorotic acid (5-FOA) [9, 10, 14]. At the *HMR* and *HML* mating type loci, these mutations cause a minor loss of silencing that can be detected by the highly sensitive CRASH (Cre-Reported Altered States of Heterochromatin) assay [12, 15]. In both assays, the deletion of *CAC1* (it encodes the largest subunit of CAF-1) synergistically reduces gene silencing in the *pol30-6* and *pol30-79* mutants, but not in *pol30-8* [9, 10, 12]. Conversely, in *pol30-8* cells the deletion of the histone chaperones *ASF1* and *HIR1* further reduce gene silencing at the telomeres while having little effect in *pol30-6* and *pol30-79* [10]. *ASF1* acts at the advancing replication forks, but such a role for *HIR1* has not been established [16, 17]. Interestingly, in *pol30-6*, *pol30-8* and *pol30-79* strains, passage of the replication fork through *HMR* is not required for the establishment of silencing [9]. These observations prompt questions on the precise mechanisms by which PCNA affects chromatin assembly and/or maintenance.

Loss of gene silencing could be attributed to a slow recovery and reassembly of heterochromatin in the wake of the replication fork or by a full epigenetic conversion of the silenced to the active state. The highly sensitive 5-FOA resistance and CRASH assays do not distinguish which one of the two underlying events leads to the loss of silencing. In this study we revisited the silencing effects of the three *pol30* mutants with a routine 5-FOA^R^ assay and a novel drug-free low-sensitivity assay [18, 19]. Our results reveal positional differences in the effects of the *pol30* mutations that lead to transient de-repression or complete epigenetic conversions at the *VIIL* sub-telomere of *S. cerevisiae*.

## Results

### *pol30* mutations lead to transient de-repression of *URA3* at the *VIIL* sub-telomere

The irreversible 5-FOA resistance and CRASH assays [12, 20] do not distinguish between events of transient destabilization of gene silencing or a complete Silent→Active (S→A) conversion of a gene. Recently, we produced two dual *adh4-URA3-HTB1*→*yEGFP*-*tel* and *adh4-URA3-yEGFP*←*HTB1*-*tel* reporters for the analyses of gene silencing at the *VIIL* telomere [18, 19]. These reporters contain the *URA3* gene followed by fusion *HTB1-yEGFP* reporter driven by the Histone H2B promoter (*HTB1*) in the orientation towards or away from the telomere, respectively (Additional file 1: Fig. S1). The proportion of cells with silenced *URA3* or *HTB1-yEGFP* can be determined as the percentage of 5-FOA resistant cells (FOA^R^, high sensitivity) or GFP-negative cells (low sensitivity), respectively [19]. Loss of gene silencing is revealed by the decrease of the percentage of 5-FOA^R^ or the proportion of GFP-negative cells in the mutant relative to *wild-type* isogenic strains. These two reporters had reproduced previously documented effects of various mutants in replication factors or histone chaperones [18, 19].

**Fig. 1:**
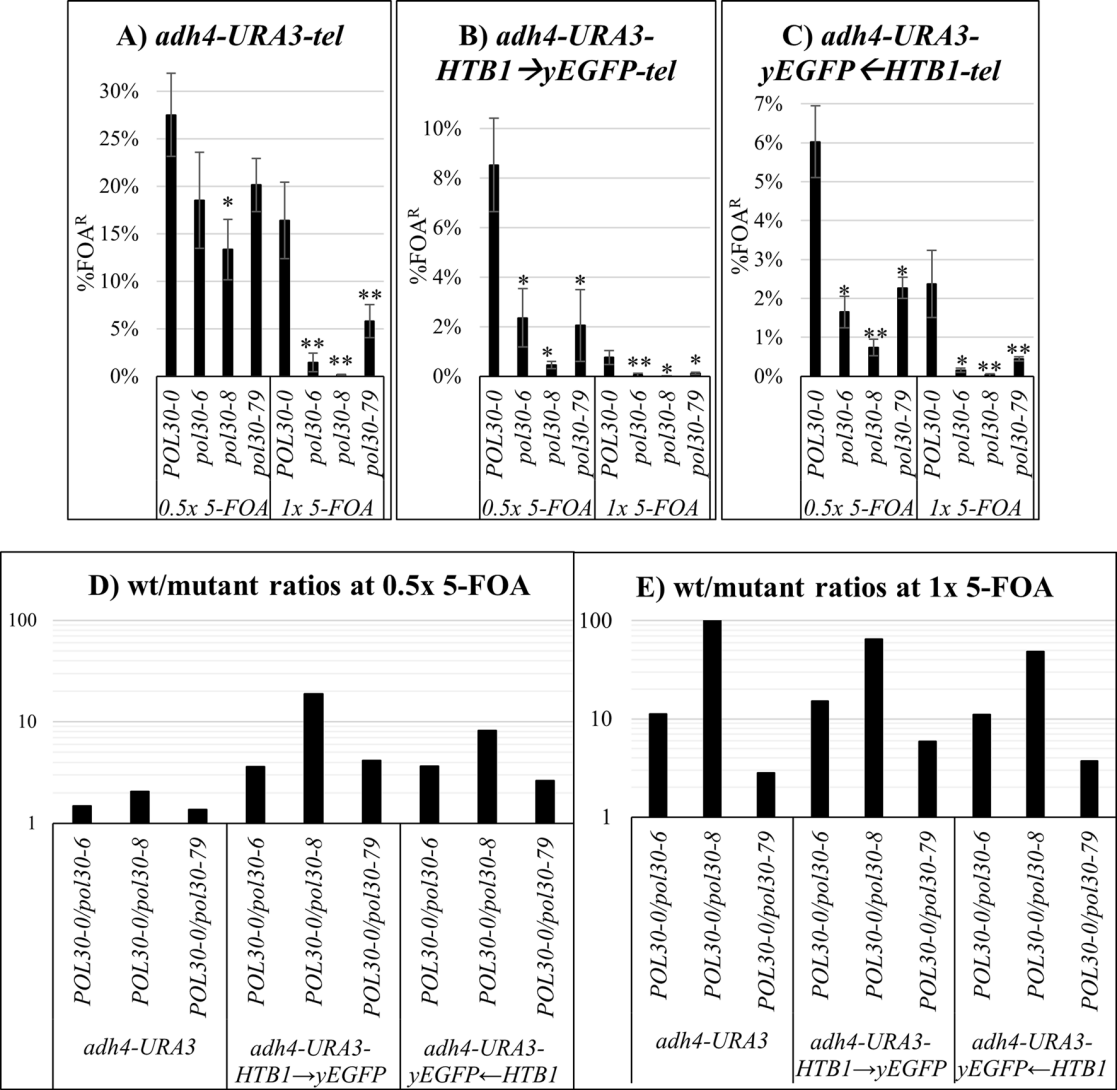
Proportions of cells with silenced *URA3* (% FOA^R^) in strains harboring *adh4-URA3-tel* (**A**), *adh4-URA3-HTB1*→*yEGFP-tel* (**B**) and *adh4-URA3-yEGFP*←*HTB1-tel* reporters. The analyses were performed with 0.5 × and 1 × concentrations of 5-FOA as described in the text. The *pol30* mutants are listed on the horizontal axis. The calculations in **A**–**C** represent average values and standard deviations s of three experiments. Asterisks represent statistical significance compared to the wild type within each concentration. *p value 0.05, **p value < 0.001. In **D** and **E** the ratio between % FOA^R^ cells in the *POL30* versus *pol30* mutant strains was calculated and plotted from the values in **A**–**C**. The logarithmic scale of the vertical axis in **D** and **E** was chosen to better capture the range of differences at the two concentrations of 5-FOA

Using the 5-FOA resistance assay, we compared the impact of the *pol30-6*, *pol30-8* and *pol30-79* alleles on the expression of *URA3* by the *adh4-URA3-tel* reporter [20], which had been used to characterize the *pol30* mutants in prior publications, and our dual reporters (Fig. 1). Two concentrations of 5-FOA were applied. We found that the proportions of FOA^R^ in the cells harboring the dual reporters were about 5 times lower that in the cells harboring *adh4-URA3-tel* (Fig. 1A–C). We attribute these differences to the longer distance between the telomere and *URA3* and of the presence of the *HTB1* promoter in-between (Additional file 1: Fig. S1). Importantly, all three reporters produced similar reductions of the percentage of FOA^R^ cells in the various *pol30* mutants (Fig. 1D, E). These results were in agreement with previous analyses of the *pol30* mutants with the FOA^R^ or the CRASH assays [9, 10, 12]. Taken together, we conclude that the dual reporters faithfully capture the effects of the *pol30* mutations.

The higher concentration of 5-FOA revealed a larger difference in the percentage of FOA^R^-cells between the mutants and the *POL30* strain (Fig. 1C). This outcome suggested that the two concentrations of 5-FOA detect a gradient of cellular *URA3* expression and not a distinct bi-modal Active/Silent state of this gene. While it was apparent that the gradient was shifted towards higher expression of *URA3* in the *pol30* mutants, the mechanism that causes this shift was not clear. We attempted to resolve this issue by flow cytometry analyses of the expression of the juxtaposed *HTB1-yEGFP* reporters.

### Lack of bi-modal active/silent expression of*** yEGFP***←***HTB1-tel***

**Fig. 2:**
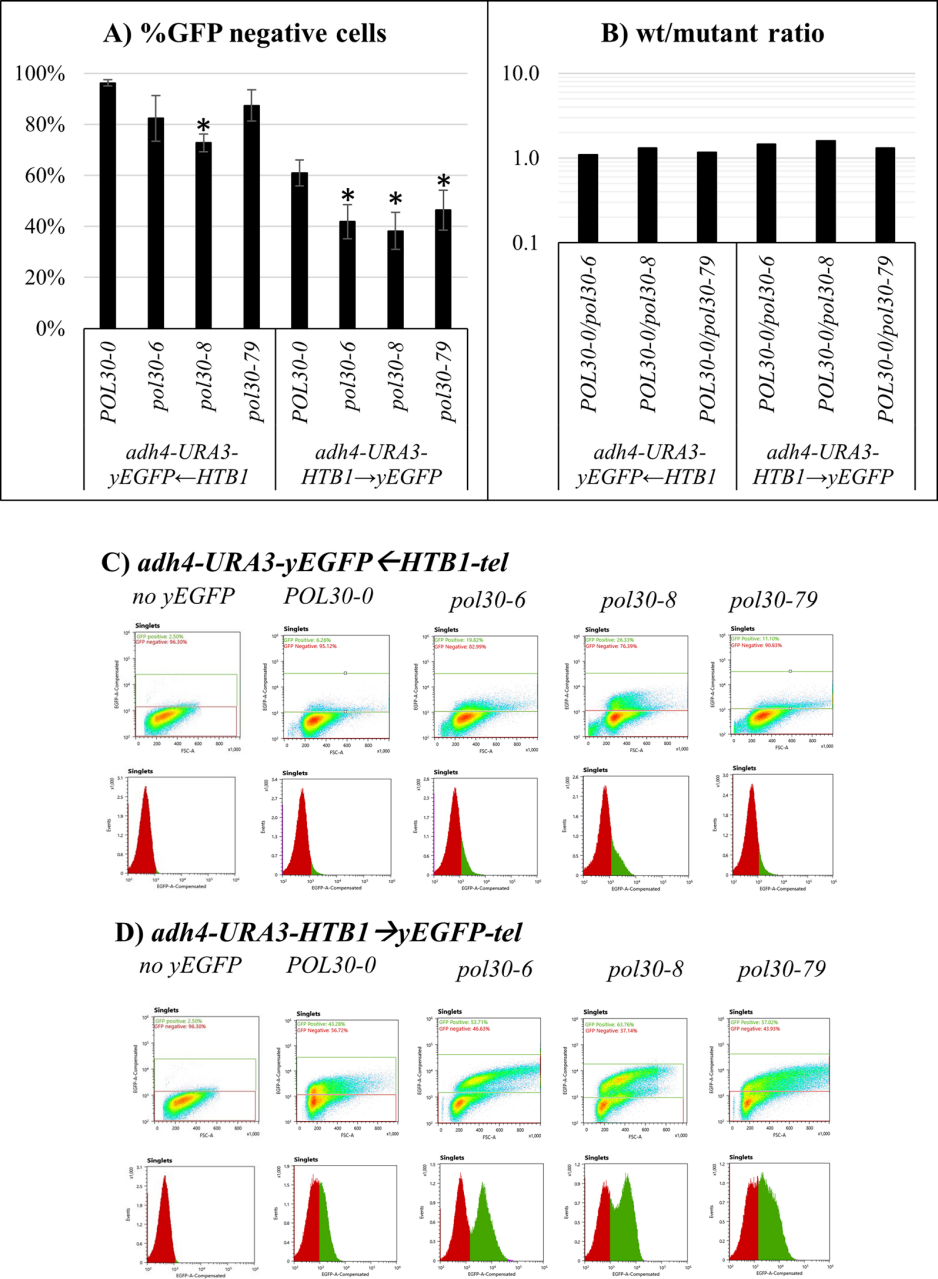
Flow cytometry analysis of cells harboring the *adh4-URA3-yEGFP*←*HTB1-tel* (**A**) and *adh4-URA3-HTB1*→*yEGFP-tel* (**B**) reporters. **A**, **B** Flow cytometry density plots (top) and GFP signal distribution graphs (bottom) with the indicated strains are shown. **C** Percentage of GFP-negative cells from three independent experiments are plotted. Asterisks represent statistical significance compared to the wild-type harboring each fragment. *p value 0.05. **D** The ratio between the proportions of GFP-negative cells in *POL30* versus the *pol30* mutants was calculated and plotted in an exponential graph

We used an isogenic *POL30* strain with no yEGFP reporter to set the upper threshold for the GFP-negative cells, which are the functional equivalent of the 5-FOA^R^ cells. Based on this criterium, the calculated percentage of GFP-negative cells harboring the *adh4-URA3-yEGFP*←*HTB1*-*tel* construct in the *pol30* mutants displayed results consistent with the results with this construct in the FOA^R^ assay; however, the proportions of GFP-negative cells were in the 75–98% range compared to 0.2–6% range of FOA^R^ cells (compare Figs. 1C and 2A). Consequently, the calculated differences between the *POL30* and the mutant *pol30* strains followed a similar trend but were significantly smaller compared to the differences observed with the FOA^R^ assay (compare Figs. 1D, E and 2B). Hence, there was a notable discrepancy in the detection of silenced reporters by the high sensitivity and low sensitivity assays. Importantly, the flow cytometry analyses revealed little effect of the *pol30* mutations on the silencing of the *yEGFP*←*HTB1*-*tel* reporter (Fig. 2C). Equally important, there was no evidence for a bi-modal Active/Silent state of *HTB1-yEGFP* expression in the *POL30-0*, *pol30-6* and *pol30-79* strains, and a tiny population of cells with elevated GFP signal in the *pol30-8* strain (Fig. 2C). These results supported the idea that the *pol30* mutations cause a transient de-repression and not a conversion to a distinct active state of *yEGFP*←*HTB1*-*tel* reporter. This transient de-repression cannot be detected by a low sensitivity assay.

### Bi-modal active/silent expression of ***HTB1***→***yEGFP-tel***

We readily observed two distinct populations of GFP-positive and GFP-negative cells when the flow cytometry assays were performed with the *adh4-URA3-HTB1*→*yEGFP*-*tel* reporter (Fig. 2D). In the *POL30* strain, the percentage of the GFP-negative cells was 60% and decreased to about 40% in the mutants. The differences between *POL30* and the mutants were far smaller when compared to the differences detected by the FOA^R^ assay with *URA3*, again supporting the idea that the FOA^R^ assay is detecting transient de-repression and not Silent→Active epigenetic conversions. Importantly, the comparison between the *adh4-URA3-HTB1*→*yEGFP*-*tel* and *adh4-URA3-yEGFP*←*HTB1*-*tel* reporters pointed to a difference in the expression of *HTB1-yEGFP* depending on the position and orientation of the reporter and independent of the effect of the *pol30* mutations (Fig. 2A, and B).

We noticed a higher spread of the GFP-positive population across the FSC-A axis. We performed a Pearson correlation analysis between FSC-A and EGFP-A compensated values for both orientations of the fragment. A linear positive correlation was observed in all strains, with strains harboring the *HTB1*→*yEGFP-tel* fragment showing stronger correlation (Additional file 1: Fig. S2A). Using microscopy, we followed up by measuring the size of the cells harboring no GFP or the *HTB1-yEGFP* fragments in both orientations. This analysis showed that all *pol30* mutants have an increased cell size as compared to the *wild-type* counterparts (Additional file 1: Fig. S2B). However, we did not observe any statistically significant difference between the strains with and without *HTB1-yEGFP*. These analyses suggest that although higher GFP signal and FSC-A are associated, the cell size and the GFP signal statistical associations are not caused by the *HTB1* promoter. Equally importantly, the single or bi-modal modes of expression of the reporters are not related to the size of the cells.

### Analyses of the expression of *HTB1-yEGFP* by fluorescent microscopy

**Fig. 3:**
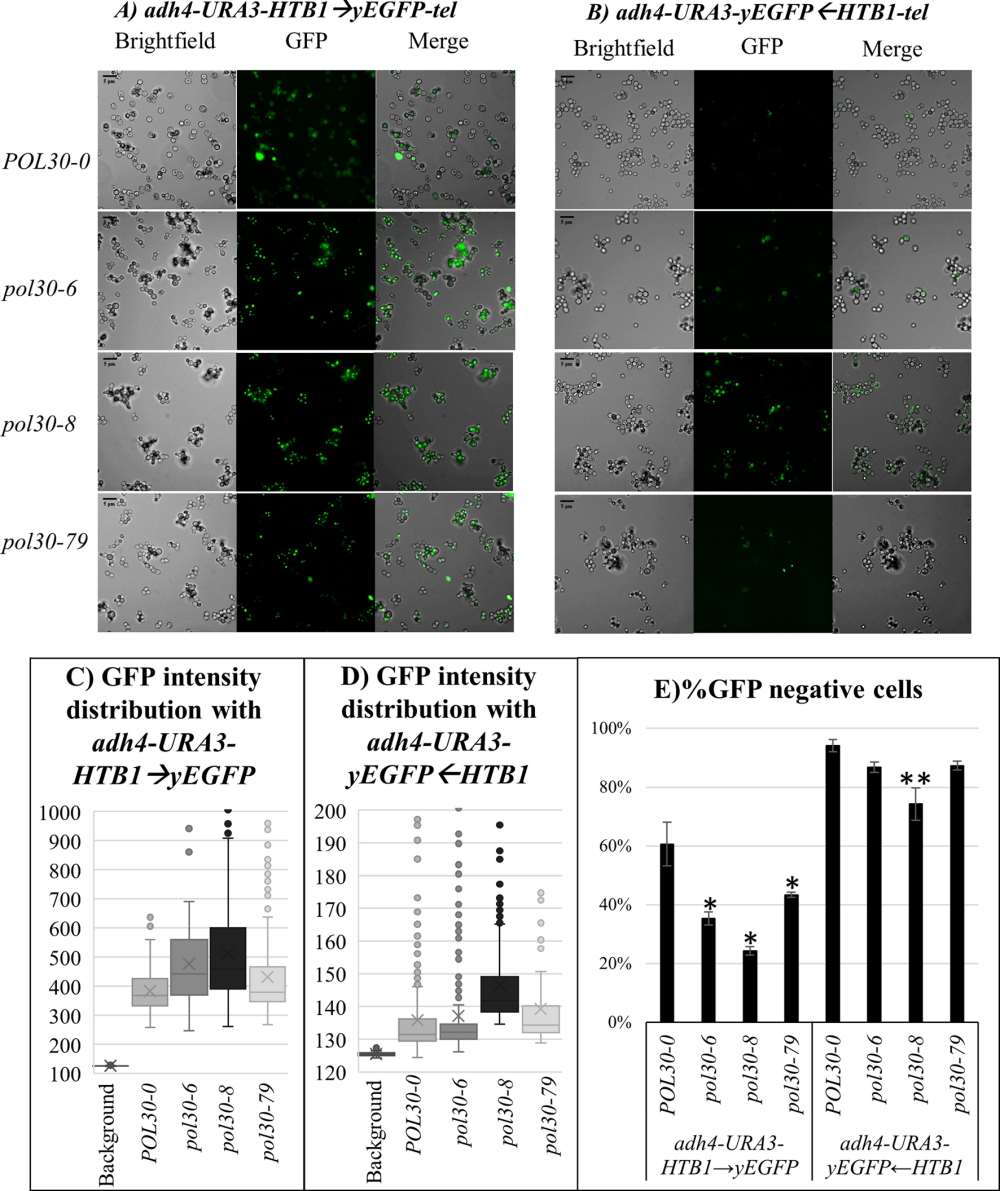
Fluorescent microscopy analysis of cells harboring the *adh4-URA3-HTB1*→*yEGFP-tel* (**A**) and *adh4-URA3-yEGFP*←*HTB1-tel* (**B**) reporters. **A**, **B** Images of indicated strains captured at ×40 resolution. While the signal ratios in the two panels were kept constant, the brightness/contrast of the GFP and MERGE images in the righthand and lefthand panels were altered to better represent the on/off state of yEGFP expression and the differences in intensity of the yEGFP signal between the two constructs. **C** GFP intensity distribution of strains harboring *adh4-URA3-HTB1*→*yEGFP-tel* fragment compared against background intensity. **D** GFP intensity distribution of strains harboring *adh4-URA3-yEGFP*←*HTB1-tel* fragment compared against background intensity. **E** Percentage of GFP-negative cells from three independent experiments are plotted. Asterisks represent statistical significance compared to the wild-type harboring each fragment. *p value 0.05, **p value < 0.001

We followed with analyses of the expression of *HTB1-yEGFP* using fluorescent microscopy (Fig. 3A, B). The measurement of the intensity of GFP fluorescence in individual cells demonstrated that the *adh4-URA3-HTB1*→*yEGFP*-*tel* construct produced, on average, 3.5 times higher signals compared to the *adh4-URA3-yEGFP*←*HTB1*-*tel* construct across all strains (Fig. 3C, D). Both constructs produced higher signals in the *pol30* mutants relative to the isogenic *POL30-0* strain with *pol30-8* cells showing the strongest effect (Fig. 3C, D). The percentage of GFP-positive cells was determined as follows. We averaged the intensity of signals in ROI (Region of Interest) with no cells and postulated a threshold of 1.25% higher signal for GFP-positive cells. By these criteria, in the *POL30-0* strain the two constructs produced 60% and 94% GFP-negative cells, respectively (Fig. 3E). The reduction of GFP-negative cells in the *pol30* mutant strains mirrored the effects observed by flow cytometry (Figs. 2C and 3E). Again, the *pol30-8* mutation showed the strongest statistically significant effect regardless of the construct used, while only the *adh4-URA3-HTB1*→*yEGFP*-*tel* construct produced statistically significant reduction of silencing in the *pol30-6* and *pol30-79* strains (Fig. 3D). These analyses were in good agreement with the data obtained by flow cytometry and 5-FOA-resistance assays (Figs. 1 and 2).

### Genetic interactions of *pol30* mutants with *CAC1* and *ASF1*

Previous studies have shown that the deletion of *CAC1* has a synergistic negative effect on the gene silencing at the *VIIL* sub-telomere in *pol30-6* and *pol30-79*, but not *pol30-8* mutants [9, 10]. Conversely, in *pol30-8* cells, the deletion of the histone chaperone *ASF1* further reduced silencing at the sub-telomeres while having little effect in *pol30-6* and *pol30-79* [10]. These studies were performed with the 5-FOA-resistance assay and did not distinguish between transient de-repression and/or epigenetic conversions of *URA3*. Therefore, we asked if the deletions of *ASF1* and *CAC1* in the *pol30* mutants would present evidence for transient de-repression or a bi-modal Active/Silent state of the *HTB1-yEGFP* reporter.

**Fig. 4:**
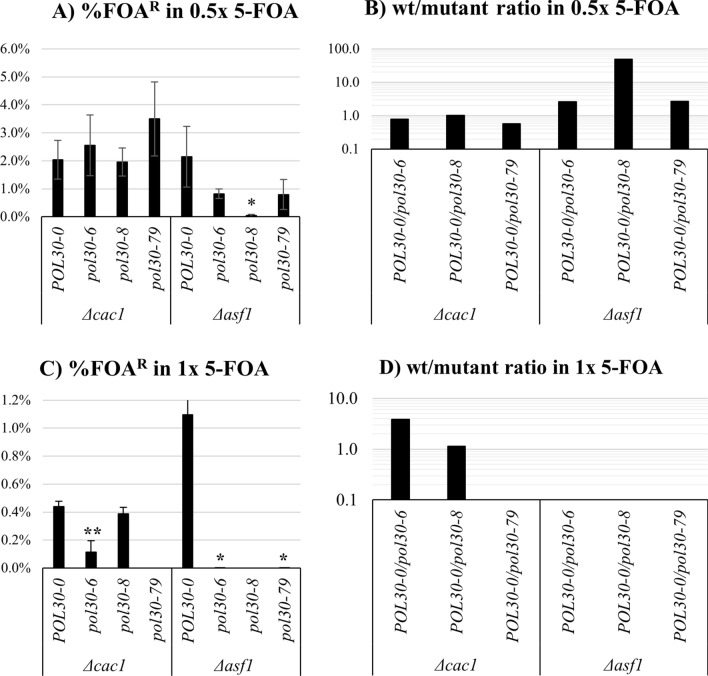
Analysis of *URA3* gene expression in mutant *pol30* strains harboring deletions of *CAC1* and *ASF1*. The analyses were performed using *adh4-URA3-yEGFP*←*HTB1-tel* fragment at 0.5 × and 1 × concentrations of 5-FOA as described in the text. The *pol30* mutants are listed on the horizontal axis. The graphs in **A**, **C** represent average values and standard deviations of three experiments at the indicated concentrations. Asterisks represent statistical significance compared to the wild type within each gene deletion background. *p value 0.05, **p value < .001. **B**, **D** The ratio between % FOA^R^ cells in the *POL30* versus the *pol30* mutant strains were calculated and plotted from the values in **A** and **C**

First, we confirmed that *URA3* in these reporters can also reproduce the previously reported observations of the *pol30* mutations in *cac1∆* and *asf1∆* genetic backgrounds. The analyses were performed with the *adh4-URA3-HTB1*←*yEGFP*-*tel* construct, which produced slightly higher percentage of FOA^R^ cells (Fig. 1C) and can more reliably detect the reduction of silencing in the *cac1∆* and *asf1∆* strains. We found that at 0.5 × 5-FOA the *pol30* mutations had no statistically significant effect in the *cac1∆* background (Fig. 4A, B). At the higher 1 × 5-FOA concentration, *pol30-6* and *pol30-79*, but not *pol30-8*, exacerbated the silencing defects at *URA3 in cac1∆* cell (Fig. 4C, D)*.* In the *asf1∆* background, the *pol30-8* mutation markedly reduced the silencing of *URA3* at both concentrations of 5-FOA (Fig. 4A, C), while *pol30-6* and *pol30-79* showed statistically significant loss of silencing only in 1 × concentration (Fig. 4C, D). Hence, our construct can reproduce the effects of *cac1∆* and *asf1∆* in the context of the three *pol30* mutations [9, 10, 14]. Notably, the effects of *cac1∆* and *asf1∆* have been reproduced at different concentrations of 5-FOA, thus reiterating that subtle differences in the concentrations of 5-FOA could lead to different interpretations in different studies.

**Fig. 5:**
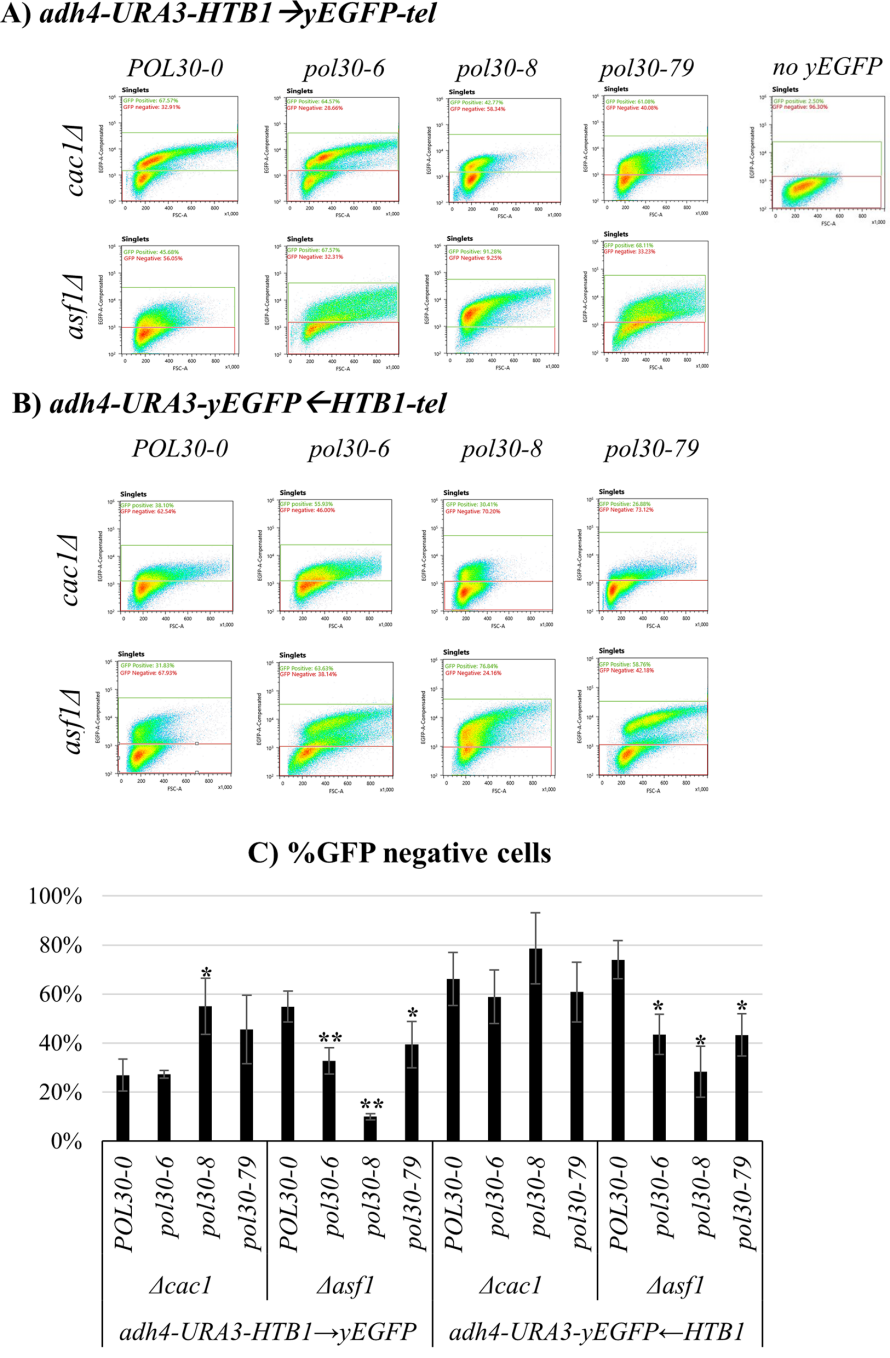
Genetic interactions of the *pol30* mutants with *CAC1* and *ASF1*. **A** Density plots of the *pol30* strains with deletions of *CAC1* and *ASF1* analyzed by the *adh4-URA3-HTB1*→*yEGFP-tel* construct. **B** Density plots of the *pol30* strains with deletions of *CAC1* and *ASF1* analyzed by the *adh4-URA3-yEGFP*←*HTB1-tel* construct. **C** The percentage of GFP-negative cells from three independent experiments with the indicated strains are plotted. Asterisks represent statistical significance compared to the wild-type harboring each fragment in each genetic background. *p value 0.05, **p value < 0.001

Next, we tested how the deletions of *CAC1* and *ASF1* affect the expression of *HTB1-yEGFP* in the two reporters. As previously shown in Fig. 2, the *adh4-URA3-HTB1*→*yEGFP*-*tel* reporter displayed a distinct bi-modal Active/Silent state of the expression of the *HTB1-yEGFP* (Fig. 5A, Additional file 1: Fig. S3A, B). The deletion of *CAC1* decreased the proportion of GFP-negative cells in all four strains, with stronger effects in the *pol30-6 and pol30-79* strains (Fig. 5C). The deletion of *ASF1* had a stronger effect in the *pol30-8* mutants (Fig. 5C).

The analyses with the *adh4-URA3-yEGFP*←*HTB1*-*tel* construct revealed a different picture. In Fig. 5B, we show that the deletion of *CAC1* in the context of *POL30* and the *pol30* mutant backgrounds decreased the overall proportions of GFP-negative cells as demonstrated by the upward shift of the signals in the density plots. However, a clear bi-modal distribution of GFP-positive and GFP-negative cells was observed only in the *pol30-8* mutant (Fig. 5B, Additional file 1: Fig. S3A, B). This, along with the data in Fig. 4, suggests that the deletion of *CAC1* only leads to transient de-repression of both genes in this dual reporter. In contrast, the deletion of *ASF1* produced a small but distinct proportion of GFP-positive cells in *POL30* cells, which was increased in the *pol30* mutants with *pol30-8* having a more significant effect (Fig. 5B, C, Additional file 1: Fig. S3C, D). We conclude that at this position of the *HTB1-yEGFP* reporter the loss CAF1 function is leading to transient de-repression and that this effect is similar to the one caused by the deficiency of *POL30* function. Conversely, the deficiency of Asf1p leads to a bi-modal Active/Silent state of the reporter. In both genetic backgrounds, the mutations in *POL30* quantitatively exacerbate these effects, but have distinct qualitative effects.

### Genetic interactions of *pol30* mutants with *RRM3*

The replication forks frequently pause in the sub-telomeric regions of the chromosomes. The events of pausing are exacerbated in *rrm3*∆ mutants, but the exact positions of the pausing are not known [21, 22]. *RRM3* encodes a DNA helicase necessary for the restart of paused replication forks [17]. We considered the possibility that the positional effects observed with our dual reporters are linked to the pausing of the fork. If this assumption is correct, the deletions of *RRM3* would have different effects on the expression of *URA3* and *HTB1-yEGFP* in the two reporters.

**Fig. 6:**
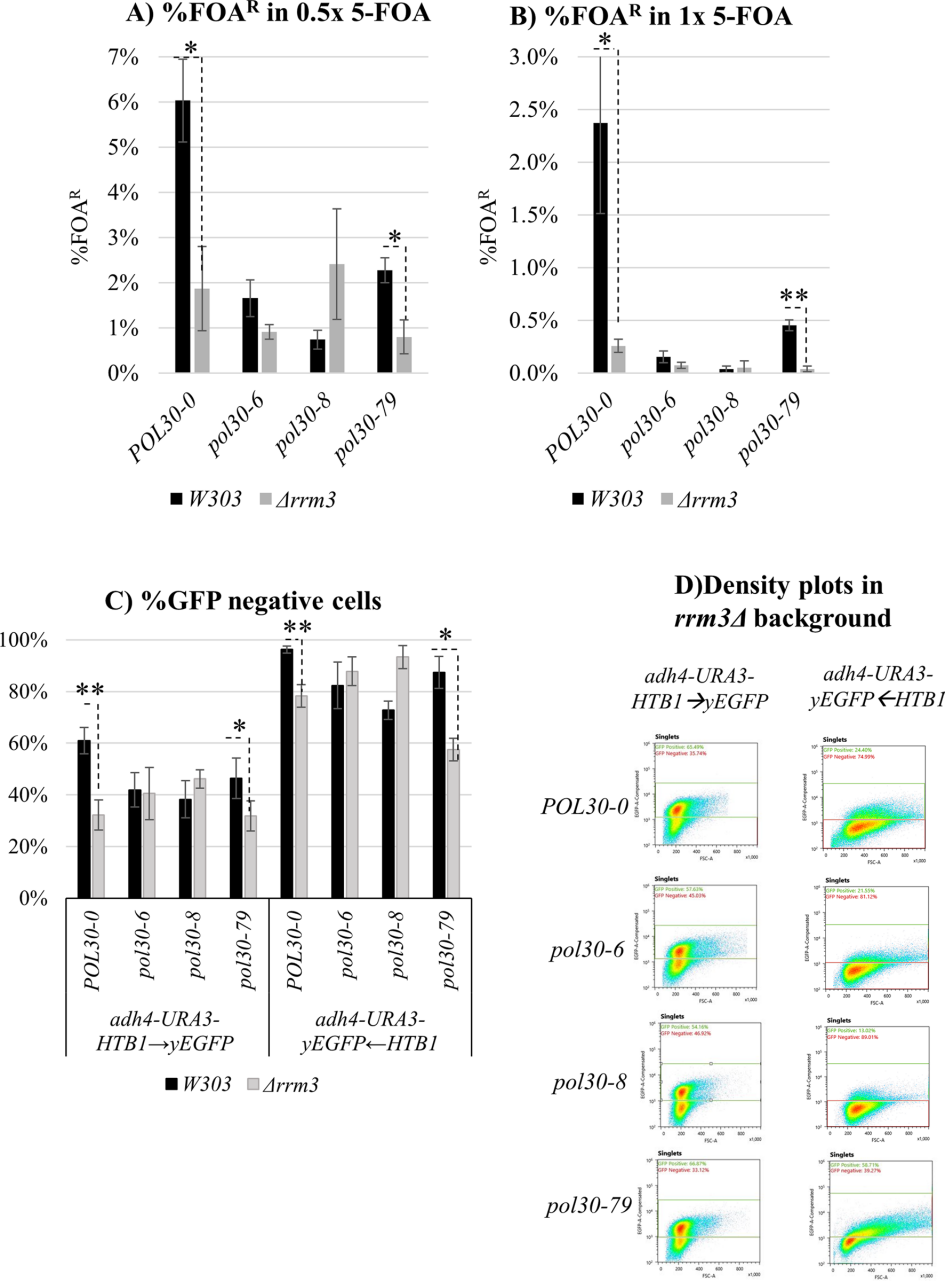
Analysis of *URA3* gene expression and genetic interactions of the *pol30* mutants with *RRM3. URA3* gene expression analyses were performed at ×0.5 (**A**) and ×1 (**B**) 5-FOA concentrations. The *pol30* mutants in *W303* and *rrm3Δ* backgrounds are listed on the horizontal axis. The calculations in **A** and **B** represent average values and standard deviations of three experiments. **C** The percentage of GFP-negative cells from three independent experiments with the indicated strains are plotted. **D** Density plots of the *pol30* strains with deletions of *RRM3* analyzed by *adh4-URA3-HTB1*→*yEGFP-tel* and *adh4-URA3-yEGFP*←*HTB1-tel* constructs. Asterisks represent statistical significance between connected strains. *p value 0.05, **p value < 0.001

In Fig. 6A, B, we show that the deletion of *RRM3* in the *POL30-0* and *pol30-79* strains reduced the percentage of FOA^R^ cell threefold to tenfold at 0.5 × or 1 × 5-FOA concentration, respectively. The deletion of *RRM3* in the *pol30-6* and *pol30-8* strains did not show a significant additive effect (Fig. 6A, B, Additional file 1: Fig. S4A). We followed up by flow cytometry to measure the expression of *HTB1-yEGFP*. In both constructs, deletion of *RRM3* produced minor, but statistically significant decreases in the percentage of GFP-negative cells in *POL30-0* and *pol30-79* strains and not in *pol30-6* and *pol30-8* strains (Fig. 6C, D Additional file 1: Fig. S4B, C). Hence, it seems unlikely that the observed positional effects in the expression of *HTB1-yEGFP* are caused by the pausing of the fork. At the same time, it is noteworthy that *RRM3* synergistically interacts with *POL30-0* and *pol30-79*, but not with the *pol30-6* and *pol30-8* alleles.

### Physical interactions of the *pol30* mutants with Cac1p and Rrm3p

**Fig. 7:**
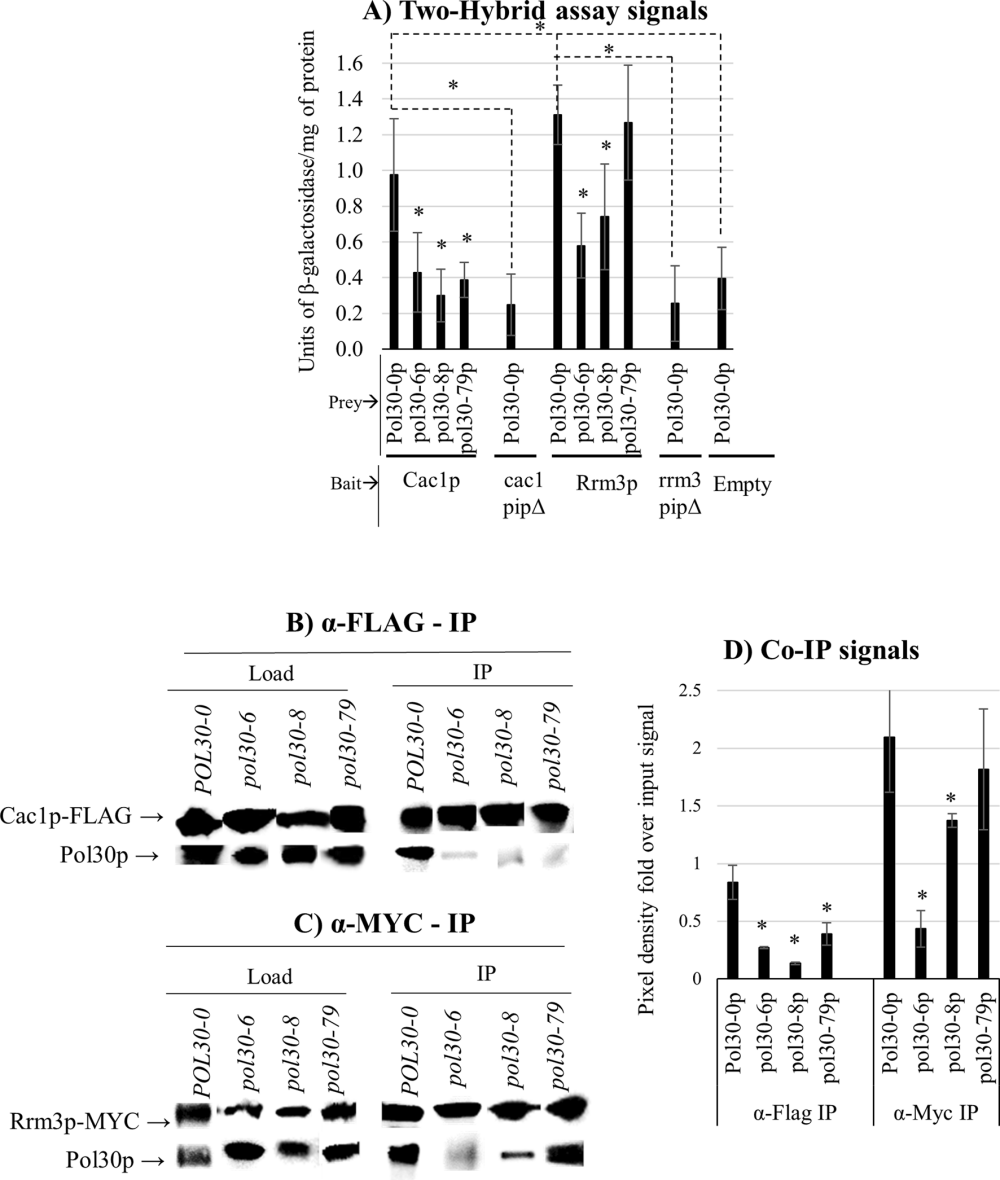
Physical interactions of the *pol30* mutant proteins with Cac1p and Rrm3p. **A** Yeast two-hybrid interaction measured as units of β-galactosidase produced per milligram of protein (U/mg of protein). Average values and standard deviations from three independent experiments are plotted on Y-axis as a factor of wild-type protein interactions. The bait and prey plasmids are indicated on the graph. **B** Co-immunoprecipitation of Pol30p and mutant pol30p by Cac1p-FLAG. Western blot was performed using α-PCNA and α-FLAG antibodies. **C** Co-immunoprecipitation of Pol30p and mutant pol30p by Rrm3p-Myc. Western blot was performed using α-PCNA and α-Myc antibodies. **D** Pixels of western blot band images were quantified, and the Pol30p bands were normalized against corresponding Cac1p/Rrm3p band intensities. Average values and standard deviations from two independent experiments with two biological replicates were plotted. Asterisks represent statistical significance compared to the wild-type Pol30p in each IP. *p value 0.05, **p value < 0.001

Cac1p and Rrm3p contain a PIP (PCNA Interacting Peptide) and both are known to directly interact with Pol30p (PCNA) proteins in vitro [7, 8, 23–25]. We tested if these two proteins differentially interact with the mutant Pol30p proteins by a two-hybrid interaction assay as described previously [25]. Cac1p and Rrm3p were used as baits and the wild type and mutant Pol30p as prey. In this assay, the three *pol30* mutations reduced the binding to Cac1p to the levels observed with the negative *cac1PIP∆* control in which the PCNA-Interacting-Peptide (PIP) sequence was destroyed (Fig. 7A). The interaction with Rrm3p was reduced by the *pol30-6* and *pol30-8* mutations, but not by the *pol30-79* mutation.

We followed up by co-immunoprecipitation assays with Cac1p-FLAG and Rrm3p-Myc tagged proteins (Fig. 7B, C). These were expressed from low copy plasmids in the *POL30-0* and the mutant *pol30* strains and the immuno-precipitates were tested for the presence of Pol30p. In agreement with the two-hybrid assay and previous reports [9], the three Pol30p mutant proteins were found to bind poorly to Cac1p-FLAG (Fig. 7B, D). The interaction of Rrm3p was reduced fivefold by the *pol30-6* mutation, less than twofold by *pol30-8* and was not affected by *pol30-79* (Fig. 7C, D). We conclude that all three mutations in *POL30* affect the association with Cac1p, while *pol30-6* and *pol30-8* reduce the association with Rrm3p.

## Discussion

### Transient loss of gene silencing in *POL30* mutants

Earlier studies have reported that mutations in *POL30* lead to loss of gene silencing at the sub-telomeric and mating type loci of *S. cerevisiae* and that different *pol30* mutations have distinct, synergistic effects with the deletion of the histone chaperones CAF-1 and Asf1p [9, 10, 12]. In this study, we used the strains from [12] and conducted a detailed analysis with three different reporter constructs and three alternative assays. In all three constructs, we tested the expression of *URA3* and reproduced the previously reported effects. However, the application of increasing concentrations of 5-FOA revealed significant differences in the proportions of 5-FOA-resistant and 5-FOA-sensitive cells (Fig. 1). These observations pointed to the possibility that the mutations in *POL30* cause transient de-repression of *URA3*. This possibility was further supported by the comparison of FOA^R^ assays data to the parallel analyses of the expression of the *HTB1-yEGFP* at two different positions in the *VIIL* sub-telomere. Indeed, the mutations in *POL30* that lead to a dramatic reduction of the percentage of 5-FOA-resistant cells reduced the percentage of GFP-negative cells at a much lower magnitude (Fig. 2). Considering the higher sensitivity of the 5-FOA resistance assay, we favor the idea that the mutations in *POL30* lead to transient de-repression of *URA3* and *HTB1-yEGFP* and not to a higher incidence of conversions from silent to active state. This outcome is in agreement with the recent analyses of the silencing of the *HML* mating type locus by CRASH assay [12]. Importantly, our results and interpretation add a significant detail to earlier studies [9, 12] and indicate that the most likely effects observed were through transient de-repression and not through the shifting of a bi-modal variegated state as previously thought.

### Position effects in sub-telomeric gene silencing

We observed qualitative differences in the expression of *HTB1-yEGFP* when the promotor of the reporter was positioned distal or proximal to the telomere. In the *HTB1*→*yEGFP-tel* orientation the reporter displayed a clear bi-modal population of GFP+/GFP− cells, while in the *yEGFP*←*HTB1-tel* orientation, the reporter produced a single population of cells with GFP signals spreading below and above the threshold of signals from cells with no reporter cells. The *pol30* mutations moderately reduced the silencing of both constructs (Fig. 2) but did not lead to a qualitative shift to a bi-modal expression in the *yEGFP*←*HTB1-tel* orientation (Figs. 2 and 3). Similarly, the deletions of *CAC1* or *RRM3* did not qualitatively shift the expression of *yEGFP*←*HTB1-tel* to a bi-modal state (Figs. 5, 6, Additional file 1: Figs. S3, S4), while the deletion of *ASF1* did (Fig. 5, Additional file 1: Fig. S3).

These results clearly indicate that distinct modes of repression of epigenetically silenced genes can operate at different positions. Numerous studies had shown that a gene can be robustly and constitutively silenced. An example for such constitutive silencing would be the *HML* and *HMR* mating type loci in *S. cerevisiae* [26]*.* Under the state of constitutive repression, various defects in cis-acting silencing factors or in the progression of replication forks can lead to transient de-repression or a bi-modal state, depending on the nature of the mutation [12, 27, 28]**.** A second mode of epigenetic silencing would be a normal bi-modal Active/Silent state. In this situation, defects in many genes engaged in chromatin maintenance and/or epigenetic transmission would affect the proportion of cells with Active or Silent state. An example for such bi-modal mode of repression would be the classical *white*^*V*^ locus in *Drosophila* [29] or some, but not all, sub-telomeric genes in *S. cerevisiae*. Finally, here we present evidence for a state in which genes are transiently de-repressed but do not display a clearcut bi-modal Active/Silent state.

The distinction between these modes of silencing is important for the interpretations of different sets of results. For example, earlier studies unveiled major discrepancies in the magnitude of sub-telomeric silencing in *S. cerevisiae* when these were assessed by genome-wide array analyses or by 5-FOA-resistance assays [30–32]. It is possible that these analyses were detecting a mixture of bi-modal and transient effects, leading to mixed conclusions on the role of various genes involved in gene silencing. Similarly, discrepancies are observed when the silencing of the mating type loci in *S. cerevisiae* is assessed by the 5-FOA-resistance or the more sensitive CRASH assay.

At this point, we do not know what mechanisms underlay the modes of silencing and the roles of various silencing factors in them. However, based on our results (Figs. 4, 5 and 6), it is tempting to speculate that *CAC1*, *POL30* and *RRM3* are all involved in the prevention of excessive transient de-repression in the wake of the replication forks. One possibility is that the activity of these three genes is necessary for the timely maturation of replicated heterochromatin. On the other hand, *ASF1* has a distinct role, and its loss can lead to bi-modal mode of expression.

### Relevance to telomere position effect on native telomeres

Our synthetic reporters are devoid of sub-telomeric elements that are normally present at the native telomeres. Hence, our assays have their limitations and do not directly address the specific mechanisms of TPE at native telomeres. Nevertheless, our findings at an engineered telomeric locus are still bearing relevance to gene silencing at native telomeres, as well as other silenced loci in the genome of this and other organisms. They also challenge multiple earlier conclusions on the effects of various mutations on gene silencing in *S. cerevisiae* and add significant details on the stability of epigenetic state, its transmission and the perceived on/off variegated transcription at the heavily studied *VIIL* telomere. Equally importantly, our manuscript corrects a substantial body of work on the effects of *POL3, CAC1* and *ASF1* mutations. These prior studies were conducted using highly sensitive irreversible assays and do not necessarily address the stability of the silenced state. Finally, we complement previous work on the differences in the repression of genes at different positions in the sub-telomeres of *S. cerevisiae* [33–35] with the notion that it remains unclear if these differences are at the level of stability or at the level of on/off bi-modal expression.

### Is the pausing of replication forks responsible for the observed position effects?

Prior studies have demonstrated that replication forks frequently pause in the sub-telomeric regions of the chromosomes, but the exact positions are not known [22, 36]. It is also known that the pausing of forks in the sub-telomeres is exacerbated by the deletion of *RRM3* [22, 36]. We reasoned that a specific pause event can contribute to the different modes of repression of *HTB1-yEGFP*. If this is the case, the deletion of *RRM3* in cells harboring the two constructs would have different effects. However, the deletion of *RRM3* did not lead to a distinct silencing of the reporters in the *wild type* and *pol30* mutant strains (Fig. 6). Therefore, we suggest that the pausing of the fork is unlikely to contribute to the differences in the repression of *HTB1-yEGFP* at the two positions.

### Physical interactions of Pol30p(PCNA) with Cac1p and Rrm3p

Previous studies have demonstrated that Pol30p directly interacts with the Cac1p subunit of CAF-1 and with Rrm3p [25]. It had also been reported that the in vitro interaction between Pol30p and Cac1p is severely diminished by the *pol30-6* and *pol30-79* mutations and to a lesser extent by the *pol30-8* mutation [9]. However, it is not known how these mutations affect the interaction with Rrm3p. We addressed this question by two-hybrid interaction and immunoprecipitation assays and reproduced the results of [9] (Fig. 7). In these assays, we obtained evidence that the *pol30-6* and *pol30-8*, but not the *pol30-79*, mutations reduce the affinity to Rrm3p (Fig. 7). On the other hand, the deletion of *RRM3* in the *pol30-79,* but not in the *pol30-6* and *pol30-8*, had a synergistic negative effect on gene silencing (Fig. 6). These results are consistent with the interpretation that the loss of silencing phenotype in *pol30-6* can be partially attributed to the impaired interaction with Rrm3p. At this point, we cannot comment on how the *pol30-8* allele relates to *RRM3*. Rrm3p remains capable of physically interacting with Pol30-8p, albeit poorly (Fig. 7), yet the deletion of *RRM3* in the *pol30-8* strain had little effect on silencing (Fig. 6). We suspect that a complex interplay between Pol30p interacting factors is responsible for this lack of effect. The *pol30-79* mutation is located on inter-domain connecting loop and has been shown to be defective in replication with *POL32* (Polδ) [37]. A recent structural analysis study has shown that all three *pol30* mutations have structural anomalies at a common surface 10 Å away from PIP-binding site [38]. The retention of Rrm3p by Pol30-79p and the additive silencing defect observed upon deletion of *RRM3* in *pol30-79* strain pose the question if Rrm3p binds to a non-canonical PIP binding site and plays an important role in maintaining fork stability in these defective strains.

#### Concluding remarks and significance

In this study, we have described two distinct modes of gene repression at the telomeres of *S. cerevisiae*: transient repression and a bi-modal Active/Silent state. We have demonstrated that mutations in *POL30*(PCNA) or the deletions of *CAC1* and *RRM3* affect the transient mode of repression, while the deletion of *ASF1* leads to the conversion from transient mode to the Active/Silent state. Future analyses of gene silencing in this and other organisms should consider these two modes of repression.

## Materials and methods

### Yeast strains, plasmids, and primers

The *POL30* and the mutant *pol30* strains are identical to the ones published in [12]. The derivatives of these strains were produced by PCR-directed deletion of the target genes as described in [19], and were PCR confirmed. The reporter fragments were inserted at the *VIIL* telomere via electroporation and were confirmed by PCR. The *adh4-URA3-tel* construct was identical to the one described in [20]*.* The *adh4-URA3-HTB1*→*yEGFP*-*tel* and *adh4-URA3-yEGFP*←*HTB1*-*tel* reporters were described in [18, 19]. All strains are listed in Additional file 2: Table S1. Primers used for knocking out genes are listed in Additional file 2: Table S2. The recombinant strains were routinely maintained in YPD medium (1% yeast extract, 2% tryptone, 2% glucose) at 30 °C. Flow cytometry and fluorescent microscopy experiments were conducted in Synthetic Complete (SC) media. Plasmids used in two hybrid assay and co-immunoprecipitation experiments are listed in Additional file 2: Table S3. Generation of the expression plasmids are described in [25]. *pol30* mutations were introduced in *pBL240-PCNA-Gal4*_*AD*_ prey plasmid using site directed mutagenesis. Primers used for site-directed mutagenesis are listed in Additional file 2: Table S2. The plasmid was amplified with partially overlapping primers harboring *pol30-6*, *pol30-8* and *pol30-79* mutations using PaCeR™ HP™ Master Mix (GeneBioSystems#PCR-002-01). The PCR products were digested with Dpn1 and transformed in *E. coli DH5α*. Plasmids extracted from *E. coli* was sequenced to confirm insertion of the mutations and were transformed in yeast strain W303 using lithium acetate transformation. Plasmids utilized in co-immunoprecipitation experiment were introduced in *POL30* and mutant *pol30* strains using electroporation. Strains for co-immunoprecipitation and two hybrid experiments were grown in Synthetic Drop-out (SD) media, as appropriate.

### 5-FOA sensitivity assay

Cells were grown in YPD at 30 °C overnight. Saturated cultures were serial-diluted by 1:10 and 5 μL of each dilution was spotted on YPD, SC-Ura, 0.5 × 5-FOA and 1 × 5-FOA plates. Final 5-FOA concentrations were 5 mg/mL in 0.5 × 5-FOA plates and 10 mg/mL in 1 × 5-FOA plates (BioBasic#703-95-7). All plates were incubated at 30 °C for 3–5 days. The colonies were counted with a Gallenkamp colony counter and the percentage of 5-FOA resistant cells were calculated and plotted using Microsoft Excel®.

### Flow cytometry

Cells were grown in synthetic complete media up to OD_600_ = 1 and washed with phosphate buffer saline. Resuspended cells were sonicated for two cycles of 30 s ON/10 s OFF with a Mandel Scientific ultrasonic sonicator at 50% output to disperse cell clusters. 488 nm laser of Sony SH800z flow cytometer was used to detect GFP, and LESH00SZFCPL*™* software was used to generate the density plots and to analyze data. 100,000 events were screened for each strain, and GFP negative gate was set based on an isogenic strain lacking GFP. Data from at least three independent experiments were pooled in Microsoft Excel® to create %GFP negative graph and calculate standard deviation.

### Fluorescent microscopy

Cells were grown in SC media and 2 µL of the cell suspension were analyzed on 8-well microscope slides. Images were captured by Leica DM600B with brightfield and green fluorescent channels at 40× magnification. Volocity™ software was used to quantify GFP intensity and number of GFP positive cells. The intensity of the GFP signal in individual cells was determined as follows. The pixel values of 100 cell-free ROI (regions of interest) were averaged and deemed background. The pixel values in ROI over at least 100 cells were then measured and plotted in distribution graph. The evaluation of the percentage of GFP+ cells was determined as described in [19]. A negative threshold was set at 125% of average background intensity and cells with intensity scores greater than the set threshold was counted as GFP positive. Images from three independent experiments were used to show the GFP distribution, calculate % GFP negative population and standard deviation.

### Yeast two-hybrid assay

Cells containing reporter, bait and prey plasmids were grown in SC/Ura^−^/Leu^−^/His^−^/2% glucose media at 30 °C up to OD_600_ 0.8–1. Cells were then collected, washed with cold H_2_O, and incubated in SC/Ura^−^/Leu^−^/His^−^/2% galactose/1% raffinose media at 30 °C for 4 h. Cells were then harvested and washed with ice-cold H_2_O and resuspended in 3 mL of buffer P (50 mM sodium phosphate, pH 7.7, 300 mM sodium acetate, 1 mM 2-Mercaptoethanol, 500 nM DTT, 10% v/v glycerol, 1 mM PMSF and 1% v/v protease inhibitor cocktail (Sigma#P8215)). The cells were split in three aliquots, pelleted, and resuspend in 200 µL Buffer P to extract protein in three replicates. 200 µL of 0.55 mm glass beads (Cole-Parmer BioSpec#11079105) were added to each tube and vortexed at 30 s on/10 s off pulse for 15 min at 4 °C. The cell lysates were centrifuged to remove debris and 50 µL of the extracts were aliquoted to measure total protein concentration. On a 96-well plate, extracts from each replicate were serially diluted by 1:10 with Buffer P and 4 mg/mL ortho-Nitrophenyl-β-galactoside (ONPG) (Sigma #N1127-1G) was added to each well. The plate was incubated at 30 °C and a timer was started immediately to record the time it takes to develop a medium-yellow color in the positive control wells. Thermo Scientific Multiskan GO plate reader was used to record the absorbance of the wells at 420 nm and 550 nm, and the time of reading was recorded. The absorbance readings were taken at three different timepoints. The units of β-galactosidase were calculated using the following formula:

$$U=\frac{1000\times \left[\left({OD}_{420}\right)-(1.75\times {OD}_{550})\right]}{(t)\times (v)\times ({OD}_{600})}$$

Total protein concentrations were measured using Bio-Rad protein assay (#500-0006) and used to normalize the β-galactosidase signal as units of β-galactosidase/mg of protein. Data from three independent experiments were pooled to calculate averages and standard deviations.

### Co-immunoprecipitation

Cells harboring Cac1p-FLAG and Rrm3p-Myc expressing plasmids were grown in SC/Leu^−^/Lys^−^/2% glucose at 30 °C up to OD_600_ 0.8–1. The cells were harvested, washed with ice-cold H_2_O and resuspended in 300 µL IP Buffer (150 mM NaCl, 50 mM Tris (pH7.5), 2 mM EDTA, 5 mM NaF, 5 mM β-Glycerophosphate, 0.1 mM NaVO3, 1% v/v protease inhibitor cocktail (Sigma#P8215) and 1 mM PMSF). 300 µL of 0.55 mm glass beads (Cole-Parmer BioSpec#11079105) were added to each tube and vortexed at 30 s on/10 s off pulse for 15 min at 4 °C. The cell lysates were centrifuged to remove debris and 50 µL of the extracts were aliquoted to load as input protein. The remaining lysate was split in half and used for α-FLAG and α-Myc pulldowns using Rat α-DYKDDDDK (Cell Signaling#14793S) and Mouse α-Myc (9E10) antibodies, respectively. The lysates were precleared with α-Rabbit/α-Mouse Ig agarose and nutated with 1ug of respective antibodies for 2 h at 4 °C. α-Flag antibody was pulled down with Protein A Sepharose beads (Invitrogen#101041), α-Myc antibody was pulled down with Protein G Sepharose beads (SinoBiological Inc#13103-PNAE-RN). The beads were washed three times with IP buffer + 0.2% Triton X100 + 0.01% SDS and eluted with 2µg of synthetic FLAG peptide (SinoBiological Inc# PP101274) or Myc peptide (SinoBiological Inc# PP100029).

### Immunoblotting

Extracts and elutes from co-immunoprecipitation were boiled in 2X Laemmli loading buffer and resolved in SDS-10% polyacrylamide gels. Proteins were subsequently transferred on polyvinylidene difluoride (PVDF) membrane using Bio-Rad Trans-Blot® SD semi-dry transfer cell at 18 V for 18 min. The membranes were blocked in TBST + 5% skim milk, cut in half, and incubated in parallel with α-FLAG/α-Myc and α-PCNA primary antibodies followed by α-rabbit/α-mouse horseradish peroxidase (HRP) conjugated secondary antibodies as appropriate. The blots were visualized using Enhanced Chemiluminescence (ECL) substrate (Bio-Rad #1705061) and imaged using Bio-Rad ChemiDoc™ XRS + with Image Lab™ software. Quantification of the signals were performed using ImageJ. PCNA band intensities in load and elute were normalized against the corresponding Cac1p/Rrm3p band intensities, and then elute intensities were expressed as a factor of corresponding input intensities. Data from at least two biological replicates were pooled to create bar graph and calculate standard deviation.

### Data analysis and statistics

Microsoft Excel® was used to calculate averages, standard deviations and to generate graphs from all experiments. Statistical analysis was performed using IBM SPSS. *P* values were determined by one-way ANOVA followed by post hoc Dunnett’s test for multiple comparisons, and by independent sample *T* test for pair-wise comparisons. *P* values < 0.05 were considered significant.

### Supplementary Information

**Additional file 1:** **Figure S1.** Schematic of the reporter fragments used in this study. **Figure S2.** Cell size analysis. **Figure S3.** GFP signal distribution by flow cytometry. **Figure S4.** Genetic interaction of *RRM3* with *pol30* mutants.

**Additional file 2:** **Table S1.** List of strains used in this study. **Table S2.** List of primers used in this study. **Table S3.** List of plasmids used in this study.

## Data Availability

All data generated and analysed in this study are included in this article and its additional files.
