# Supplementary material for: Analyses of POL30 (PCNA) reveal positional effects in transient repression or bi-modal active/silent state at the sub-telomeres of S. cerevisiae
Source: Epigenetics Chromatin. 2023 Oct 19;16:40. doi: 10.1186/s13072-023-00513-7 (PMC10585736; doi:10.1186/s13072-023-00513-7)

Supplemental Figure 1. Schematic of the reporter fragments used in this study.

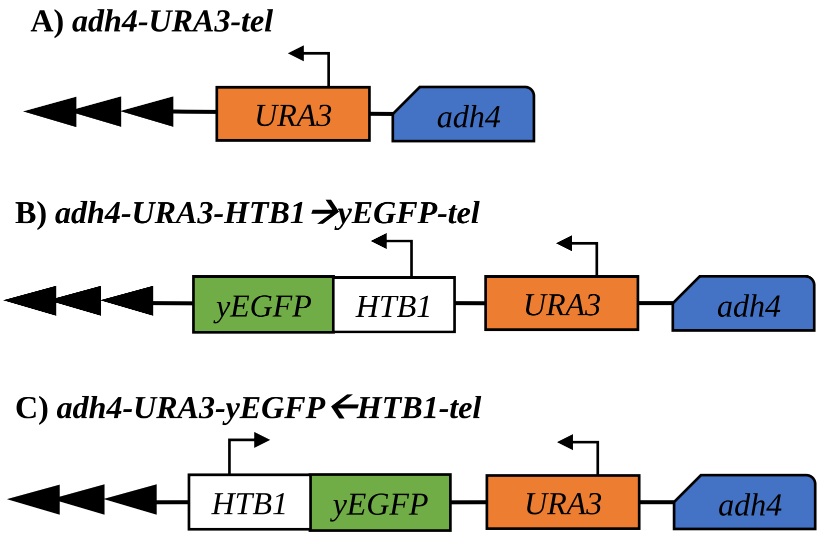

Supplemental figure 2. Cell size analysis. A) Pearson correlation coefficient heatmap between FSC-A and EGFP-compensated by flow cytometry. Correlation analysis was performed in RStudio using data of 100,000 events of strains shown in figure 2C and D and plotted as a heatmap using ggplot2 package. B) Cell size distribution using microscopy. Micrographs shown in figure 3A and B were analyzed using ImageJ software. Regions of Interest (ROI) were drawn around 100 cells for each strain and area of the cells were measured. Scale for measurement was set at 6.25pixel/µm as determined by Velocity™ imaging software. Area of the individual cells were plotted as box plot by RStudio using ggplot2 package, and multiple comparison Dunnet's test was performed using DescTools package.

A) Pearson correlation coefficient heatmap between FSC-A and EGFP

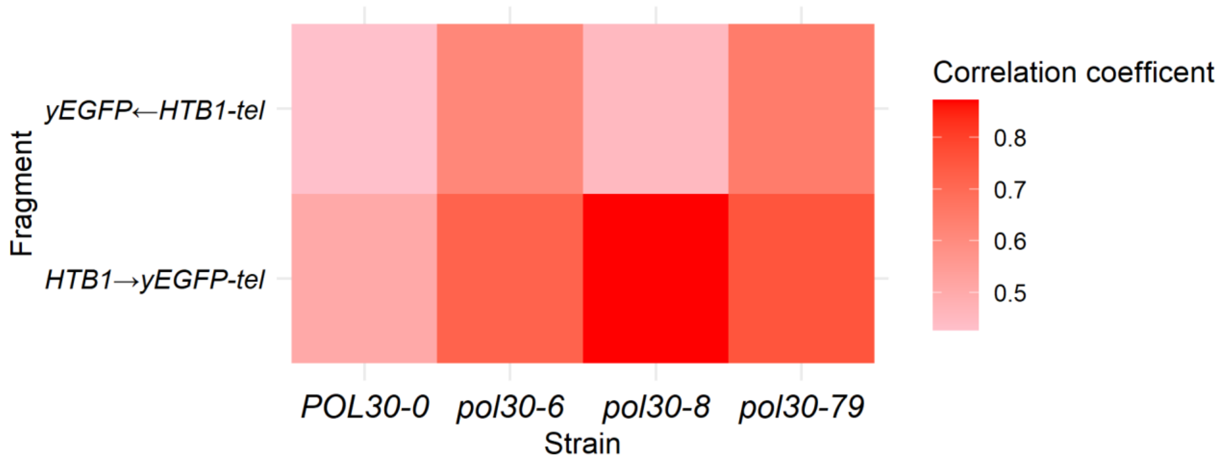

B) Cell size distribution using microscopy

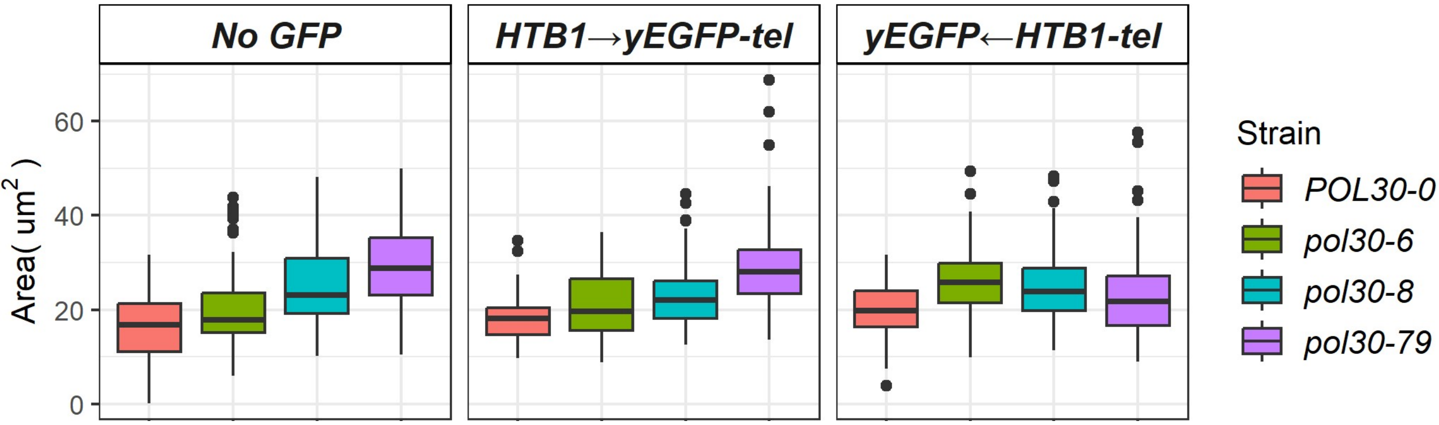

Supplemental Figure 3. ***yEGFP*** **signal distribution by flow cytometry** using A) *adh4-URA3-HTB1→yEGFP-tel* B) *adh4-URA3-yEGFP←HTB1-tel* fragments. Wild-type to mutant %yEGFP-negative ratio using both fragments are shown in log scale in C) *cac1Δ* and D) *asf1Δ* backgrounds.

A) *adh4-URA3-HTB1→yEGFP-tel*

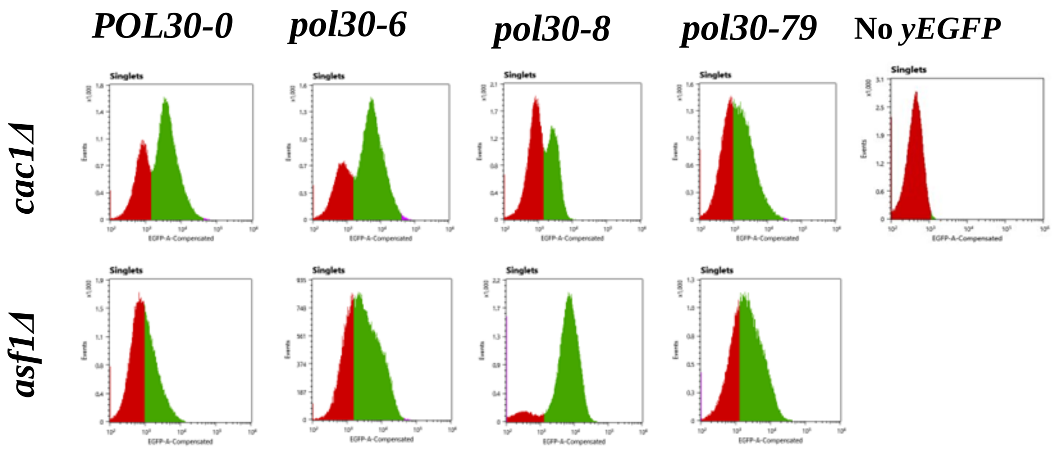

B) *adh4-URA3-yEGFP←HTB1-tel*

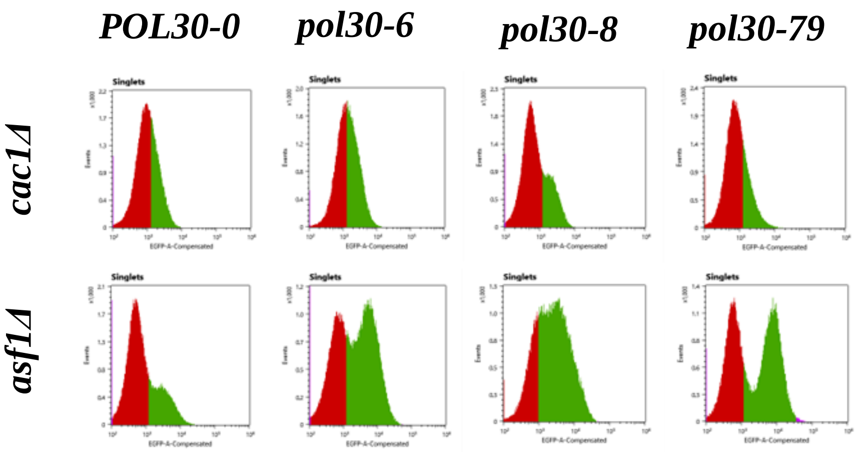

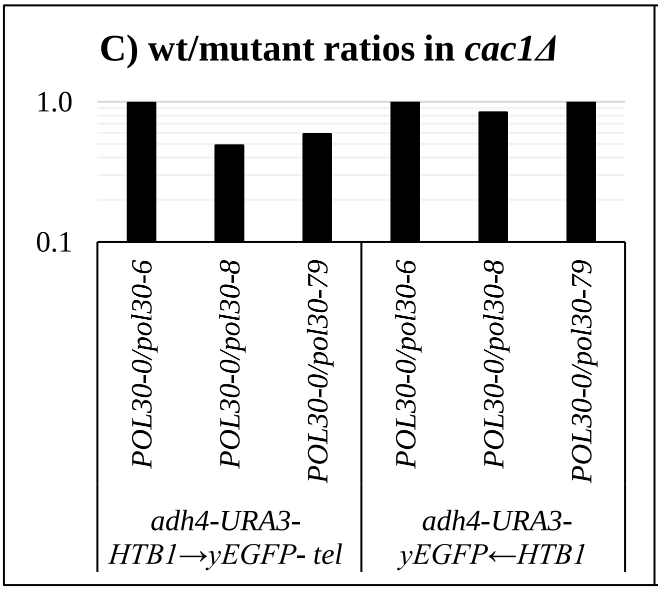

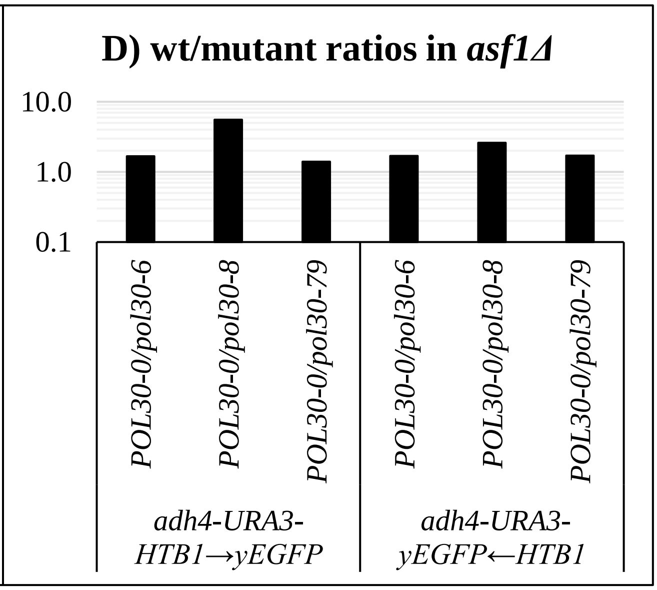

Supplemental Figure 4. Genetic interaction of *RRM3* with p*ol30* mutants. Wt/mutant ratio of A) %FOA^R and B) %GFP negative cells are shown in *rrm3Δ* background on a log scale. C) yEGFP flow cytometry distribution image with both fragments in *rrm3Δ* strains

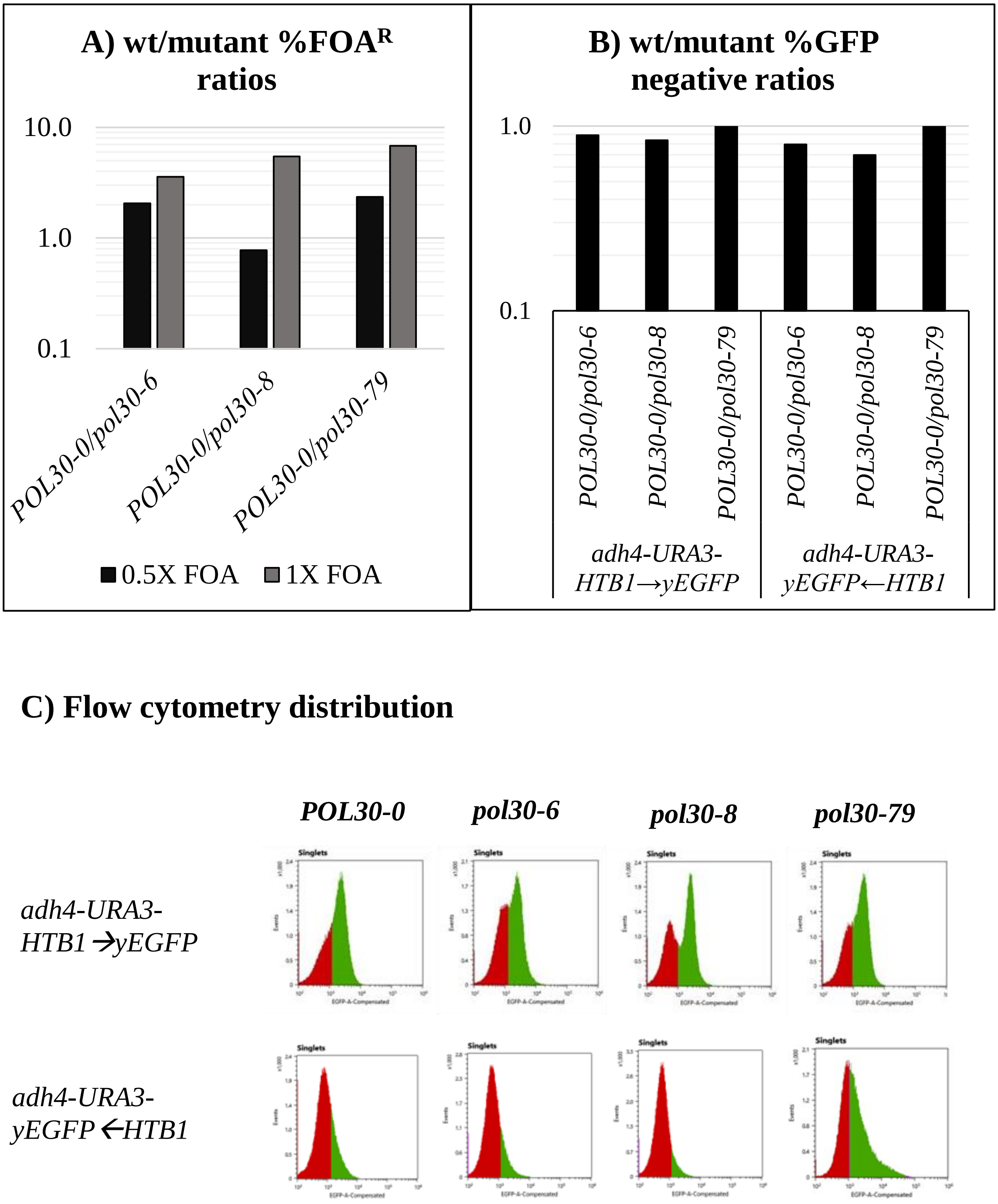

Supplement: Supplementary file 1 — Additional file 1: Figure S1. Schematic of the reporter fragments used in this study. Figure S2. Cell size analysis. Figure S3. GFP signal distribution by flow cytometry. Figure S4. Genetic interaction of RRM3 with pol30 mutants. [file 13072_2023_513_MOESM1_ESM.pdf]
